# Bilateral Humerus Midshaft Fracture Associated With Birth Trauma: A Case Report

**DOI:** 10.7759/cureus.59767

**Published:** 2024-05-06

**Authors:** Swaroop Solunke, Rahul Agrawal, Ashwin Deshmukh, Abhishek Nair, Ankit Barosani

**Affiliations:** 1 Orthopaedics, Dr. D. Y. Patil Medical College, Hospital and Research Centre, Dr. D. Y. Patil Vidyapeeth (Deemed to Be University), Pune, IND

**Keywords:** diagnosis, splints, humerus fracture, birth trauma, neonatal care

## Abstract

Bilateral humerus fractures as a result of birth trauma are a rare occurrence in neonatal care, necessitating special consideration due to their potential long-term implications. Birth-related injuries involving neonatal skeletal structures, especially fractures of the humerus, require special attention and a comprehensive approach to diagnosis and management. Here, we present the case of a newborn female child who experienced bilateral humerus fractures due to birth trauma. The subsequent management involved the application of splints to immobilize the affected arms, a standard practice in the treatment of fractures.

## Introduction

Birth trauma is a broad term encompassing various injuries that a newborn may experience during the delivery process [1]. Both maternal and fetal factors contribute to the multifactorial etiology of these injuries. A variety of mechanisms, including difficult deliveries, shoulder dystocia, fetal presentation, or the use of obstetric interventions such as forceps or vacuum extraction, have been shown to be significant neonatal and mechanistic factors [2]. Maternal factors such as gestational diabetes, macrosomia, and prolonged labor may contribute to an increased risk of birth trauma, including bilateral humerus fractures [3,4].

Albeit infrequent, fractures associated with birth trauma can still occur, with the humerus being one of the bones susceptible to such injuries [5]. In most cases, humerus fractures in newborns are unilateral. However, when they occur bilaterally, it adds complexity to the clinical scenario, necessitating a comprehensive understanding of the underlying causes and factors [6]. This understanding is crucial for developing appropriate therapeutic approaches to address the unique challenges presented by bilateral humerus fractures in newborns.

Clinical presentation plays a crucial role in the early identification of neonates with bilateral humerus fractures. The infant may exhibit signs of distress, asymmetrical movement of the upper extremities, or refusal to move the arms. Radiological evaluation, particularly X-rays, serves as a primary diagnostic modality to confirm the presence and extent of fractures. Additionally, it aids in distinguishing bilateral humerus fractures from other birth-related injuries.

## Case presentation

**Figure 1:**
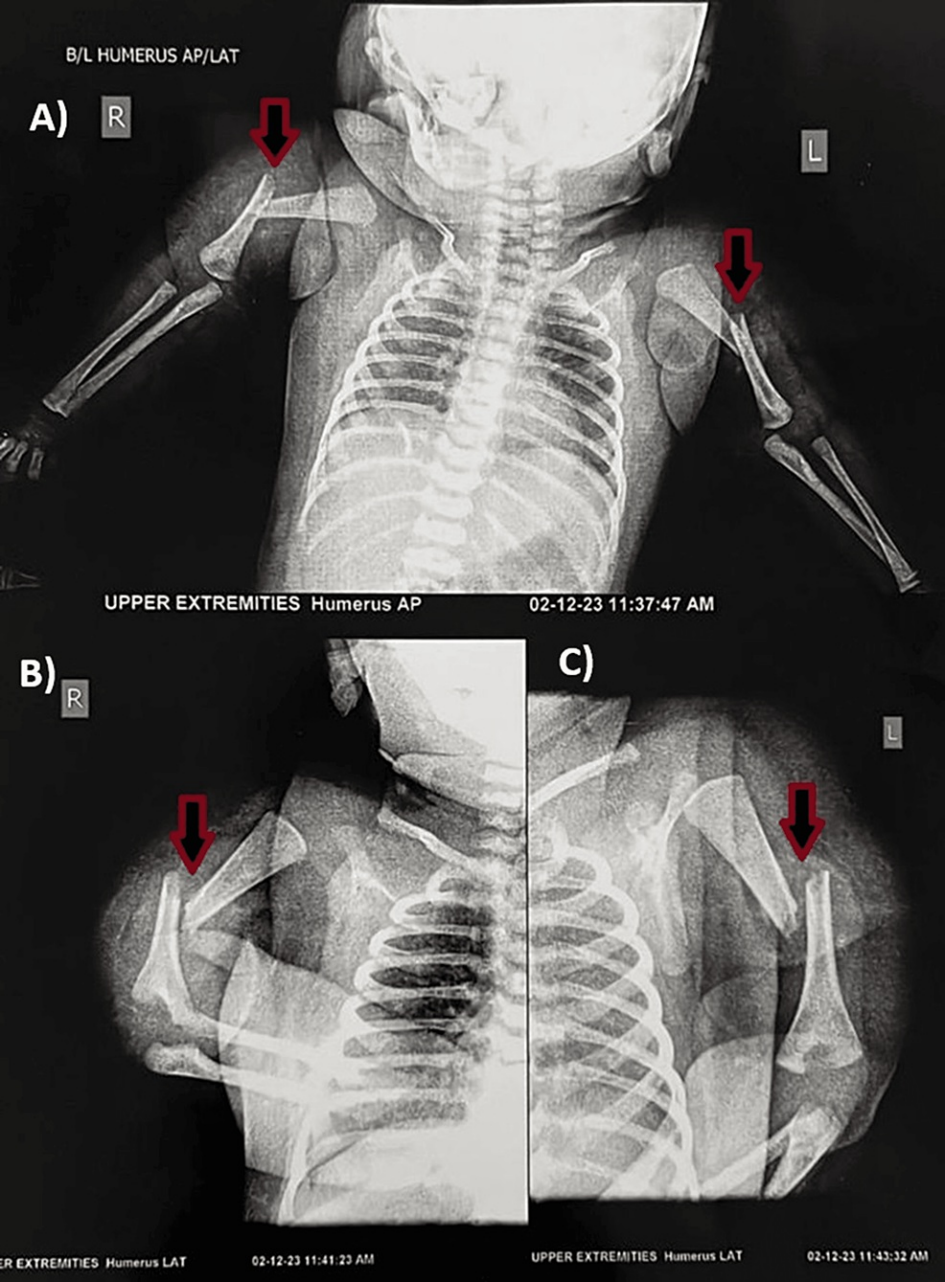
X-ray confirming bilateral humerus midshaft fracture A) Bilateral humerus midshaft fracture anterior-posterior (AP) view; B) right humerus midshaft fracture lateral view; C) left humerus midshaft fracture lateral view

The newborn female child, weighing 3.4 kilograms, was delivered at full term via a lower segment cesarian section (LSCS). The decision for LSCS was based on significant indications of fetal distress, obstructed labor, and breech presentation. Traction applied during the LSCS was noted to be forceful, attributable to the aforementioned underlying conditions. The newborn cried immediately post-birth, with appearance, pulse, grimace, activity, and respiration (APGAR) scores of 8/10 at one minute and 9/10 at five minutes. General and systemic examinations revealed no notable abnormalities. While no external congenital anomalies were observed, abnormal and discomforting movements of the upper limbs prompted orthopedic evaluation on the fifth day of life. Subsequent X-ray investigations revealed bilateral humerus midshaft fractures (Figure 1).

**Figure 2:**
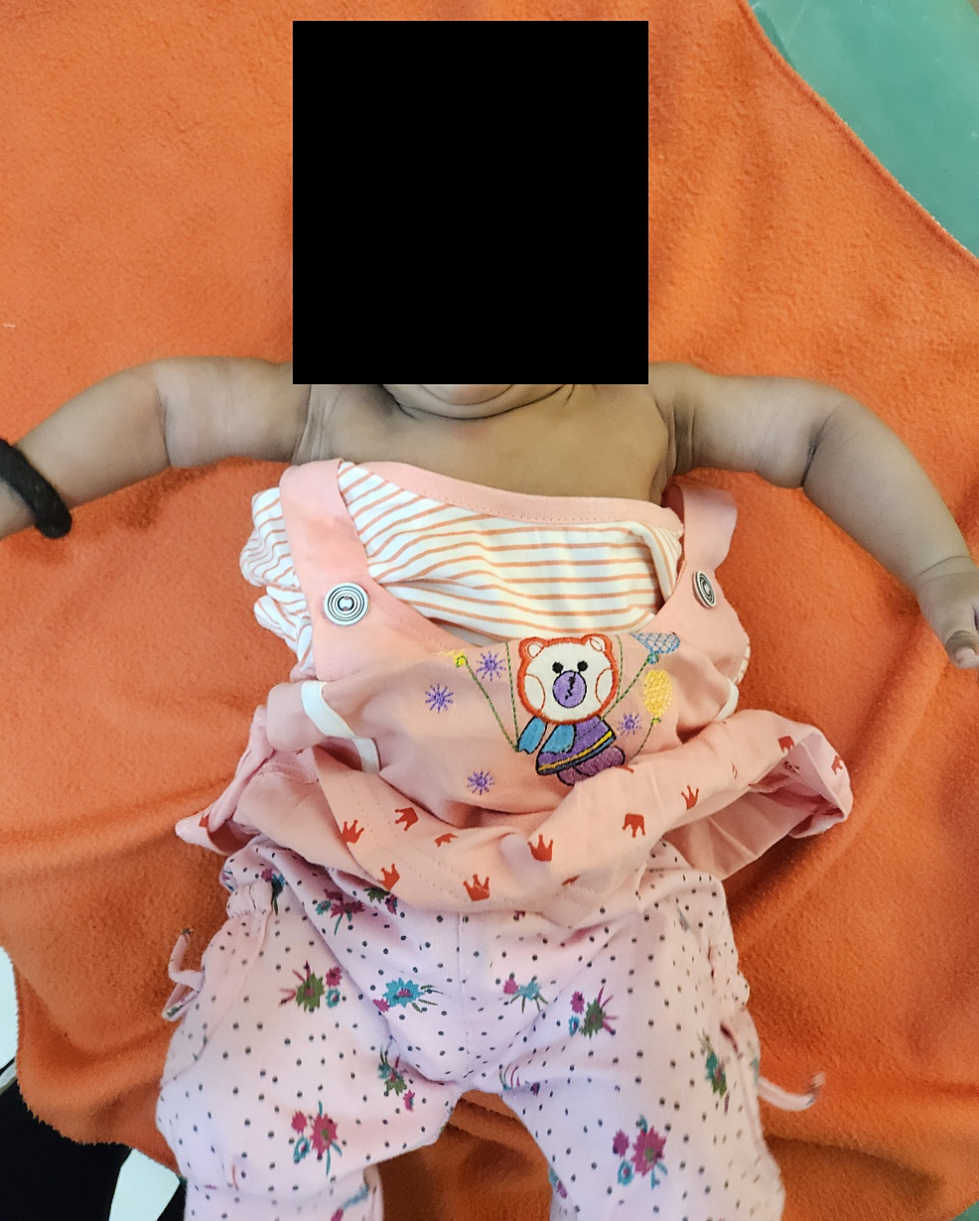
Clinical picture of the patient at the 10-week follow-up

**Figure 3:**
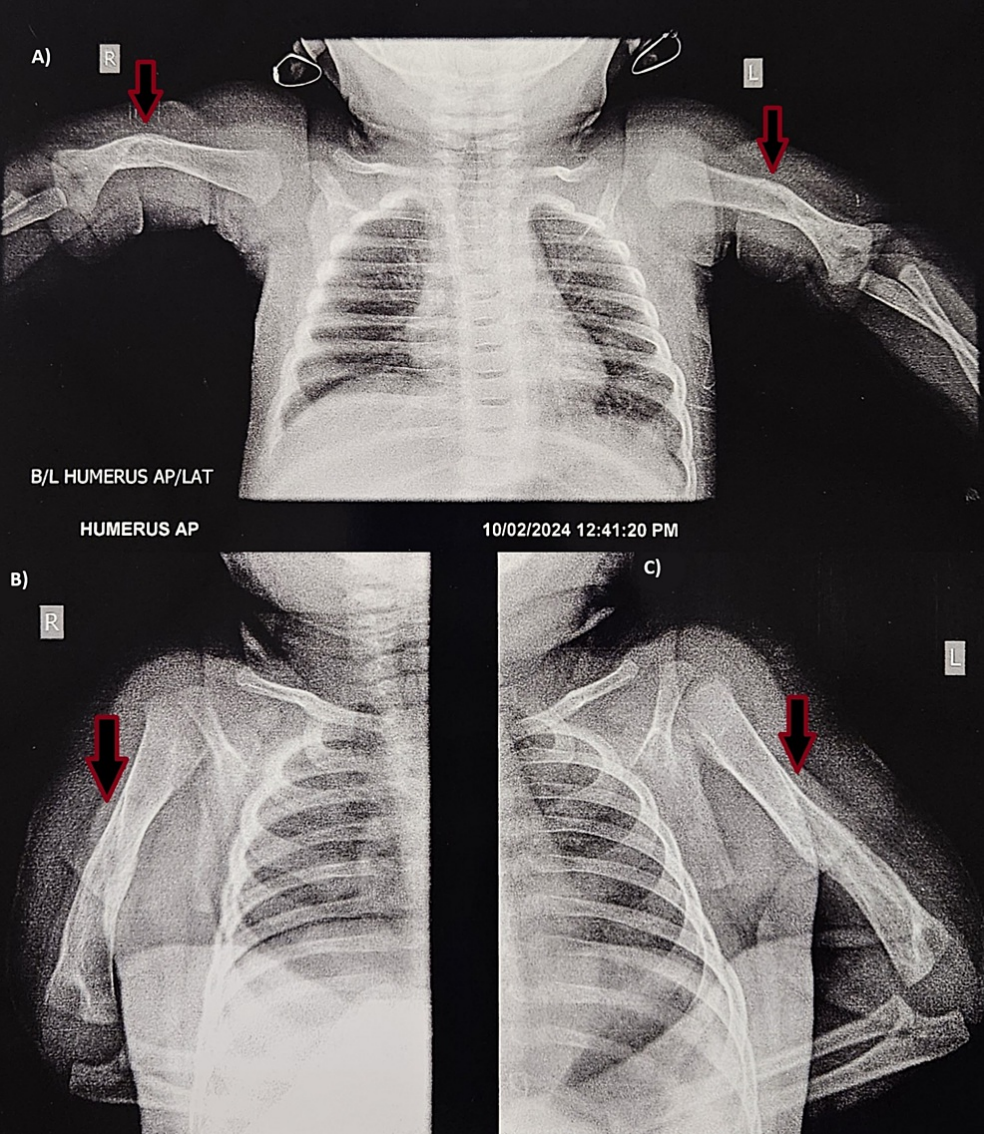
X-ray showing recovery A) Bilateral humerus anterior-posterior (AP) view; B) right humerus lateral view; C) left humerus lateral view

Immobilization of the arms was achieved through bilateral splinting, followed by strapping from the upper arm to the chest. Notably, the maternal history revealed that the mother had a confirmed case of HIV infection. Consequently, the infant was initiated on syrup nevirapine at a dosage of 1.5 mL once daily. The infant commenced on WSF (Wati spoon feed) at a full feed volume of 50 mL. Upon discharge, the patient exhibited satisfactory feeding, as well as normal bowel and bladder function. The patient was scheduled for follow-up after two weeks. However, the patient arrived after 10 weeks and could move the upper limbs comfortably (Figure 2). The X-ray investigation at the 10-week follow-up confirmed the successful recovery of the fracture (Figure 3).

## Discussion

Injuries associated with birth trauma have been linked to various factors, including obstructed deliveries, shoulder dystocia, breech fetal presentation, and the use of obstetric interventions such as forceps or vacuum extraction [7]. According to a study conducted by Awari et al. in 2003, the majority of birth traumas occur during vaginal delivery and are comparatively less common during cesarean sections [11]. The study also found that long bone fractures accounted for 4% of all reported birth injuries, indicating that skeletal injuries involving long bones as a result of birth traumas are rare [8]. Various reports have linked humerus fractures at birth to breech presentation and obstetric maneuvers performed during such deliveries [5,9].

The clinical presentation is crucial for identifying bilateral humerus fractures in neonates. Infants may display signs of distress, asymmetrical movement of the upper limbs, and hesitancy or discomfort when moving their arms [6]. Radiological evaluation remains the primary diagnostic modality for confirming the presence and extent of bilateral humerus fractures.

Management strategies for bilateral humerus fractures in neonates involve a multidisciplinary approach. Non-surgical interventions, such as immobilization with splints and strapping, are frequently employed. Surgical options may be considered in specific cases where non-operative measures are unsatisfactory [10]. Treatment aims to alleviate pain, prevent complications, and promote optimal healing of the fractures.

## Conclusions

A bilateral humerus fracture following birth trauma is a rare but significant occurrence in neonatal care. There is limited data available on bilateral humerus fractures following birth trauma in neonates. This case illustrates the challenges associated with such injuries and highlights the importance of a comprehensive and multidisciplinary approach to ensure the well-being of affected neonates. While the existing studies provide valuable insights into the epidemiology, etiology, clinical presentation, diagnostic approaches, and management strategies, further research and increased awareness of this condition will contribute to improved outcomes for affected neonates and enhance preventive measures during childbirth.
